# Cotinine: Pharmacologically Active Metabolite of Nicotine and Neural Mechanisms for Its Actions

**DOI:** 10.3389/fnbeh.2021.758252

**Published:** 2021-10-21

**Authors:** Xiaoying Tan, Kent Vrana, Zheng-Ming Ding

**Affiliations:** ^1^Department of Anesthesiology & Perioperative Medicine, and Pharmacology, Pennsylvania State University College of Medicine, Hershey, PA, United States; ^2^Department of Pharmacology, Pennsylvania State University College of Medicine, Hershey, PA, United States

**Keywords:** behavior, cotinine, neuropharmacology, nicotine, nicotinic acetylcholine receptor

## Abstract

Tobacco use disorder continues to be a leading public health issue and cause of premature death in the United States. Nicotine is considered as the major tobacco alkaloid causing addiction through its actions on nicotinic acetylcholine receptors (nAChRs). Current pharmacotherapies targeting nicotine’s effects produce only modest effectiveness in promoting cessation, highlighting the critical need for a better understanding of mechanisms of nicotine addiction to inform future treatments. There is growing interest in identifying potential contributions of non-nicotine components to tobacco reinforcement. Cotinine is a minor alkaloid, but the major metabolite of nicotine that can act as a weak agonist of nAChRs. Accumulating evidence indicates that cotinine produces diverse effects and may contribute to effects of nicotine. In this review, we summarize findings implicating cotinine as a neuroactive metabolite of nicotine and discuss available evidence regarding potential mechanisms underlying its effects. Preclinical findings reveal that cotinine crosses the blood brain barrier and interacts with both nAChRs and non-nAChRs in the nervous system, and produces neuropharmacological and behavioral effects. Clinical studies suggest that cotinine is psychoactive in humans. However, reviewing evidence regarding mechanisms underlying effects of cotinine provides a mixed picture with a lack of consensus. Therefore, more research is warranted in order to provide better insight into the actions of cotinine and its contribution to tobacco addiction.

## Introduction

Cigarette smoking remains to be a leading public health issue. Despite a steady decline over the past decades, smoking rate remained at 17.2 percent in people aged 12 or older in 2018 in the United States (Substance Abuse and Mental Health Services Administration [SAMHSA], 2019). In addition, an estimated 3.6 million middle and high school students were current users of electronic cigarettes in 2020 in the United States, posing additional risk to youth (Wang et al., 2020a). Relapse rates are high in smokers; approximately 55% adult smokers made quit attempts, but only 7.5% successfully quit smoking in 2018 (Creamer et al., 2019). Nicotine is widely accepted as the major addictive component in cigarette, and it mainly activates nicotinic acetylcholine receptors (nAChRs) to produce its reinforcing and rewarding effects (Prochaska and Benowitz, 2016). Pharmacotherapies targeting effects of nicotine (e.g., nicotine replacement therapy and varenicline) have been approved to aid in smoking cessation, but only produced modest effectiveness in promoting abstinence (Rosen et al., 2018). Therefore, there is a remaining need for better understanding of mechanisms underlying nicotine addiction and tobacco smoking.

**FIGURE 1:**
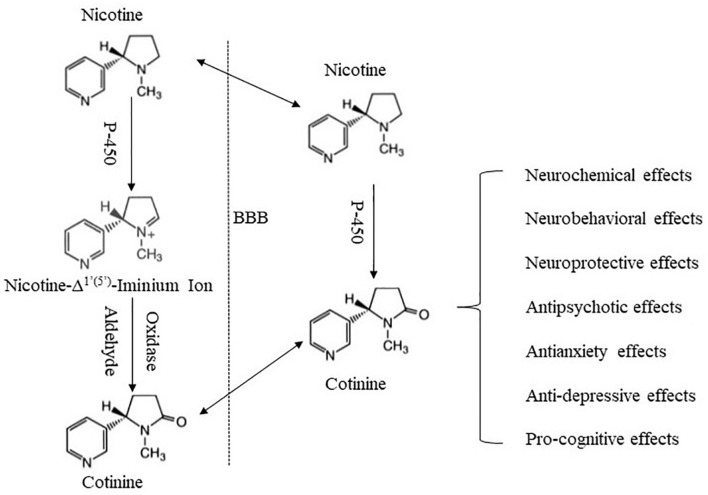
Schematic summary of cotinine formation and its major effects. Cotinine is formed mainly in the periphery via an enzyme-mediated process. Following its formation, cotinine penetrates the BBB and enters the brain. Cotinine in the brain may also be derived from *in situ* metabolism of nicotine. Cotinine then interacts with the brain to produce a variety of effects.

There are growing efforts investigating the potential involvement of minor tobacco alkaloids and nicotine metabolites in nicotine’s effects and tobacco use (Crooks and Dwoskin, 1997; Hoffman and Evans, 2013). Cotinine is a minor tobacco alkaloid and the major metabolite of nicotine. It is most commonly used as a biomarker for nicotine exposure (Benowitz and Jacob, 1994; Zhu et al., 2013). It is safe and well tolerated in humans with short-term exposure (Bowman and McKennis, 1962; Hatsukami et al., 1997), and much less toxic in rodents than nicotine (Borzelleca et al., 1962; Riah et al., 1999). Since an early study revealed cotinine’s behavioral and physiological effects (Yamamoto and Domino, 1965), accumulating evidence indicates that cotinine produces diverse effects across multiple systems, including the nervous system (Fuxe et al., 1979; Dwoskin et al., 1999), cardiovascular system (Dominiak et al., 1985; Chahine et al., 1996), endocrine system (Barbieri et al., 1989; Sofikitis et al., 2000), immune system (Rehani et al., 2008; Li et al., 2021), as well as neurobehavioral systems (Risner et al., 1985; Buccafusco and Terry, 2003). In addition, there is recognition that cotinine may contribute to some effects of nicotine (Crooks and Dwoskin, 1997; Majdi et al., 2019), raising the possibility that cotinine may play a role in tobacco use, abuse, and dependence. Herein, we review findings supporting cotinine as a neuroactive metabolite of nicotine, and discuss potential mechanisms underlying its effects. The focus is on cotinine interactions with the nervous system, and on neuropharmacological and behavioral effects of cotinine (Figure 1).

## Origin of Cotinine

Cotinine is one of the minor tobacco alkaloids which include nornicotine, anabasine, anatabine and others in tobacco products. These minor alkaloids account for approximately 5% of total tobacco alkaloids, and nicotine makes up the remainder (Benowitz et al., 1983a; Leete, 1983). Cotinine was shown to form in small quantities in fermented tobacco leaves during the tobacco curing and aging processes after harvesting, potentially through chemical oxidation of and/or bacterial actions of nicotine (Frankenburg and Vaitekunas, 1957; Wada et al., 1959). Minimal biosynthesis of cotinine was found in the living *Nicotiana Glauca* plant with negligible conversion of nicotine to cotinine (Leete and Chedekel, 1974). Cotinine is also found in other plant specifies, e.g., Carica papaya and Cestrum nocturnum (Leete, 1983). For each cigarette smoked, cotinine was absorbed in the range of 9-57 μg, far less than that of nicotine at 0.8-3 mg (Schmeltz and Hoffmann, 1977; Benowitz and Jacob III, 1984, Benowitz and Jacob, 1994; Gori and Lynch, 1985).

Cotinine, however, is the predominant metabolite of nicotine in humans and animals through enzyme-mediated oxidation of nicotine (Bowman et al., 1959; Hucker et al., 1959). Early work indicated that this enzymatic process mainly occurred in the liver involving a two-step reaction. Nicotine was first converted to 5′-hydroxynicotine by an enzyme system requiring triphosphopyridine nucleotide and O_2_, and then 5′-hydroxynicotine was oxidized to cotinine by an aldehyde oxidase (Hucker et al., 1959, 1960). An important discrete intermediate during this process was later identified as nicotine-Δ^1^′ ^(5^′^)^-iminium ion, which was catalyzed from nicotine by a cytochrome P450 (CYP)-linked oxidase, and was in rapid equilibrium with 5′-hydroxynicotine (Murphy, 1973; Brandänge and Lindblom, 1979b; Peterson et al., 1987). The oxidation of the intermediate to cotinine was demonstrated in mouse liver microsomes to also be mediated by aldehyde oxidase (Hill et al., 1972; Gorrod and Hibberd, 1982). The aldehyde oxidase was also referred to as “iminium oxidase” and was shown to exhibit high affinity for nicotine-Δ^1^′ ^(5^′^)^-iminium ion (Brandänge and Lindblom, 1979a). Subsequent research determined CYP2A6 to be the major enzyme responsible for nicotine conversion to nicotine-Δ^1^′ ^(5^′^)^-iminium ion (Cashman et al., 1992; Nakajima et al., 1996). In addition to the liver, there is evidence suggesting the metabolism of nicotine and formation of cotinine in the brain, mechanisms of which remain less clear (Jacob et al., 1997).

## Pharmacokinetics of Cotinine

In humans, an average of 70-80% of absorbed nicotine was converted to cotinine (Benowitz and Jacob, 1994; Zhu et al., 2013). Blood cotinine levels in regular smokers typically range between 250 and 350 ng/ml (1.4–2.0 μM), but can reach 800–900 ng/ml (4.5–5.0 μM) in some heavy smokers, greatly exceeding typical blood nicotine levels in the range of 10–50 ng/ml (0.06–0.3 μM) (Benowitz et al., 1983a; Benowitz and Jacob, 1994; Geng et al., 1995; Schneider et al., 2001). Oral administration of cotinine resulted in rapid absorption leading to peak systemic cotinine levels within 45 min. Bioavailability exceeded 95% following oral administration, suggesting minimal first-pass metabolism of cotinine (De Schepper et al., 1987). This is in contrast to approximate 70% of first-pass metabolism of nicotine (Matta et al., 2007). The steady-state volume of distribution was 0.7–1.0 L/kg for cotinine and 2.6–2.8 L/kg for nicotine. Plasma clearance was 0.4–1.0 ml/min/kg for cotinine and 16–17 ml/min/kg for nicotine. The elimination half-life of cotinine ranged from 12 to 16 h, in contrast to 2–2.5 h for nicotine (Benowitz et al., 1983b; De Schepper et al., 1987; Curvall et al., 1990; Benowitz and Jacob, 1994; Zevin et al., 2000; Zhu et al., 2013). The half-life of cotinine, derived from nicotine, can be up to 19–20 h, longer than that of cotinine administered as cotinine, possibly due to slow release of nicotine from tissue to blood (Benowitz et al., 1983b; Benowitz and Jacob, 1994; Zevin et al., 1997). Chronic smoking appeared to reduce the clearance half-life of cotinine (Kyerematen et al., 1982). Approximately 10–12% of administered cotinine was excreted unchanged in the urine (De Schepper et al., 1987; Curvall et al., 1990). Neither nicotine conversion to cotinine nor cotinine elimination appeared to be different between men and women (Benowitz and Jacob, 1994; Zhu et al., 2013). Plasma protein binding of cotinine was concentration-independent and averaged at 2–3%. Blood and plasma cotinine ratio averaged at 0.88, and the ratio between red blood cell and unbound plasma cotinine concentration averaged at 0.74 (Benowitz et al., 1983b). Cotinine did not seem to alter nicotine disposition or metabolism, nor was it converted back to nicotine (Keenan et al., 1994; Zevin et al., 1997; Hatsukami et al., 1998b).

In rats, approximately 60% of absorbed nicotine was converted to cotinine (Hucker et al., 1960). The half-life of cotinine formation ranged from 0.33 to 0.46 h, and maximal plasma cotinine concentrations were reached about 1.5 h after intravenous bolus administration of nicotine (Adir et al., 1976; Miller et al., 1977). Steady-state volume of distribution was 0.7–1.5 L/kg for cotinine and 2.0–5.0 L/kg for nicotine. Plasma clearance was 2.5–4.4 L/h/kg for nicotine and 0.12–0.21 L/h/kg for cotinine (Adir et al., 1976; Miller et al., 1977; Li et al., 2015). The clearance half-lives were about 5.0–9.0 h for cotinine and 20–70 min for nicotine, both of which were slightly longer in adult than early adolescent rats (Miller et al., 1977; Kyerematen et al., 1988; Sastry et al., 1995; Craig et al., 2014). Approximately 17–18% cotinine and 10–11% nicotine were excreted in urine in its unchanged form (Miller et al., 1977).

In mice, blood cotinine peaked within 10 min after intraperitoneal injection of nicotine (Petersen et al., 1984), and clearance half-life was in the range of 20–40 min, longer than that of nicotine at 6–7 min, respectively (Thompson et al., 1982; Petersen et al., 1984). Exposure to smoke from commercial cigarettes showed slowed cotinine peak time at ∼120 min and cotinine half-life could be up to ∼80 min (El Mubarak et al., 2020). Cotinine formation and elimination in mice appeared to be strain-dependent and to be influenced by mouse genotypes. For example, DBA/2Ibg mice attained 1.5 fold higher blood cotinine levels and 60-80% longer half-life than C57BL/6Ibg and C3H/2Ibg mice (Petersen et al., 1984). In combination, these disparate facts combine to demonstrate that nicotine is rapidly converted to cotinine that then is slowly removed. As a result, the body is exposed to high concentrations of cotinine for a prolonged period of time.

## Blood Brain Barrier Penetration of Cotinine

A wealth of evidence indicates that cotinine can penetrate BLOOD BRAIN BARRIER (BBB) and enter the brain. Early studies, using whole-body radiography, reported that intravenous injection of radiolabeled nicotine resulted in uniform and diffuse cotinine-related radioactivity in the brain of mice and cats, and that cotinine could be isolated from brain tissue in mice (Appelgren et al., 1962; Schmiterlöw et al., 1967). A later study indicated that brain uptake of cotinine in mice was brain region dependent with greater cotinine levels detected in cerebral cortex and basal ganglia than in hippocampus or cerebellar cortex following systemic injection of nicotine (Essman, 1973). In addition, the time-course of cotinine penetration of BBB was influenced by routes of nicotine administration. Intravenous injection of nicotine led to rapid detection of cotinine in the brain within 2-5 min, with peak levels detected 10-20 min post-injection (Stålhandske, 1970; Petersen et al., 1984; Sastry et al., 1995). Subcutaneous administration of nicotine resulted in detection of cotinine as the major metabolite in the brain at 15-30 min post-injection, which peaked around 4 h, and remained detectable 18 h after nicotine administration (Crooks et al., 1995, 1997, Katner et al., 2015). A microdialysis study showed that cotinine was detected approximately 45 min following intra-gastric administration of nicotine, and continued to increase during the 125-min collection period in the nucleus accumbens, a central reward zone (Katner et al., 2015). Cotinine accumulated in the brain following chronic administration of nicotine via an osmotic minipump in rats, with brain levels lower than serum levels (Oliver et al., 2007). Chronic nicotine exposure did not alter cotinine penetration of BBB in rats (Lockman et al., 2005).

Studies with direct cotinine administration confirmed cotinine penetration of BBB. An early autoradiography study performed in mice observed highest radioactivity in the dense cell area of the cerebellum following intravenous administration of cotinine (Bowman et al., 1964). Another study suggested that cotinine uptake into the brain was relatively homogenous with minimal regional differences (Lockman et al., 2005). In rats, subcutaneous administration of cotinine induced time- and dose-dependent accumulation of cotinine in the brain. Cotinine started to accumulate in the brain within 5 min, reached maximal levels at 20-60 min, and then gradually decreased over time with significant levels of cotinine still detected in the brain at 18 h post-administration (Crooks et al., 1997; Riah et al., 1998). In addition, subcutaneous and intravenous administration resulted in more efficient cotinine penetration than intraperitoneal administration (Riah et al., 1998). No cotinine metabolite was detected following cotinine administration, suggesting little or no biotransformation of cotinine in the brain (Crooks et al., 1997). These studies indicate that cotinine readily crosses the BBB. However, it can’t be excluded that nicotine may undergo *in situ* metabolism in the brain, thus contributing to cotinine accumulation in the brain following peripheral nicotine administration (Jacob et al., 1997).

Cotinine appears to be less efficient than nicotine in crossing BBB. In contrast to the widespread distribution of nicotine in the mouse brain, cotinine did not concentrate in the brain nearly as well (Hansson and Schmiterlow, 1962; Schmiterlöw and Hansson, 1962; Bowman et al., 1964). The brain uptake of nicotine was approximately 10 times greater than that of cotinine (Lockman et al., 2005), and an approximately 10 times higher dose of cotinine than nicotine was required to produce comparable concentrations in rats (Riah et al., 1998). In addition, the peak brain/plasma ratio for cotinine was 0.26, much lower than the 0.65 ratio for nicotine (Reavill et al., 1990; Riah et al., 1998). A human positron emission tomography study reported much lower uptake of cotinine than nicotine, in an approximately 1:6 ratio, into the frontal cortex of healthy non-smokers (Halldin et al., 1992). Nicotine is a tertiary amine, and its un-ionized form is highly lipophilic, whereas cotinine is more polar and less lipophilic (Halldin et al., 1992; Crooks and Dwoskin, 1997; Herzig et al., 1998). In addition, nicotine is transported as a mono-protonated cation across the BBB by organic cationic transport systems, whereas no active transport system has been reported for cotinine (Majdi et al., 2019). These differences in passive diffusion and active transport may contribute to the lower penetration of BBB by cotinine as compared to that of nicotine.

Brain half-lives of cotinine were 20–30 min in mice, and ∼350 min in rats, significantly longer than those of nicotine in mice at 6–7 min and in rats at ∼50–90 min (Petersen et al., 1984; Sastry et al., 1995; Ghosheh et al., 1999; Craig et al., 2014). It was estimated that, for average plasma cotinine levels at 250–350 ng/ml, the influx rate of cotinine through BBB was 0.5–0.7 ng per second per gram brain tissue, which was ∼40% of the nicotine influx estimated with average nicotine levels at 40–50 ng/ml, suggesting that cotinine may penetrate the BBB to a significant degree that would allow central actions (Lockman et al., 2005). This is consistent with evidence demonstrating cotinine’s neuropharmacological and behavioral effects in animals (Goldberg et al., 1989; Crooks and Dwoskin, 1997; Terry et al., 2005), and psychoactive effects in humans (Keenan et al., 1994; Hatsukami et al., 1998b).

## Pharmacodynamics of Cotinine

Cotinine appears to be a weak agonist of nAChRs, but there are substantial discrepancies in the literature regarding its potency (Table 1). In rat brain membrane preparations, two studies reported that Ki values of cotinine for displacing [^3^H]nicotine or [^3^H]epibatidine binding were ∼1–4 μM, and Ki values for nicotine were ∼5–15 nM, with cotinine being ∼200–250 fold less potent than nicotine (Abood et al., 1981; Vainio and Tuominen, 2001). These Ki values of cotinine are within the range of blood cotinine concentrations attained in human smokers (Hukkanen et al., 2005). Other studies reported that the potency of cotinine for displacing [^3^H]nicotine binding was 1-3 mM, and the potency of nicotine was 0.6–200 nM for nicotine, with cotinine being ∼10,000 to ∼1.5 million fold less potent than nicotine (Sloan et al., 1984; Anderson and Arneric, 1994; Riah et al., 1999). The potency of cotinine is greatly higher than physiological levels of cotinine in smokers (Hukkanen et al., 2005). Ki values for displacing [^3^H]cytisine binding (presumably high-affinity α4β2^∗^ subtype; the ^∗^ denotes other nAChR subunits) were over 200 μM for cotinine and 0.6 nM for nicotine (Anderson and Arneric, 1994). Consistently, cotinine up to 1 μM produced minimal effect on [^3^H]cytisine binding, whereas nicotine induced over 70% inhibition of [^3^H]cytisine binding in rat cerebral cortex preparations (Sziraki et al., 1999). Cotinine and nicotine were reported to display equal efficacy in displacing [^125^I]α-bungarotoxin binding (presumably low-affinity α7 nAChRs), but cotinine was ∼100 fold less potent than nicotine, with IC_50_ values at 1 mM and 10 μM, respectively (Riah et al., 1999).

**TABLE 1:** Summary of receptor binding and agonistic potency of cotinine for nAChRs and specific subtypes.

Receptor	Test system	Potency (μM)		References
		(−)-Cotinine	(−)-Nicotine	
nAChRs	Rat brain membrane	1–2	0.006–0.01	Abood et al., 1981
		3–4	0.011–0.016	Vainio and Tuominen, 2001
		> 1,000	0.0006	Anderson and Arneric, 1994
		2,000	0.2	Riah et al., 1999
		2,800	0.03	Sloan et al., 1984
	Torpedo membrane	520	0.3	Abood et al., 1981
		200,000	500	Riah et al., 1999
	Bovine chromaffin cells	130–310	0.3–1.6	Vainio and Tuominen, 2001
α4β2*	Rat brain membrane	> 200	0.0006	Anderson and Arneric, 1994
	Monkey striatal synaptosomes	65–79	0.008	O’Leary et al., 2008
	Chinese hamster ovary cells	85	0.8	Alijevic et al., 2020
α7	Rat brain membrane	1,000	10	Riah et al., 1999
	Torpedo membrane	50	25	Riah et al., 1999
	Xenopus oocytes (α7V274T mutant)	70	0.94	Briggs et al., 1999
α3/α6β2*	Monkey striatal synaptosomes	3.1–3.5	0.006	O’Leary et al., 2008

*Nicotine data, when available in the same studies, are included for comparisons.*

In squirrel monkey preparations, cotinine inhibited ^125^I-α-conotoxinMII (a ligand for α3/α6β2^∗^ nAChRs) binding in the caudate with an IC_50_ value of ∼3.5 μM, which was ∼600-fold less potent than nicotine at 5.7 nM. Cotinine also inhibited [^125^I]A-85380 (a ligand for both α3/α6β2^∗^ and α4β2^∗^ nAChRs) binding with an IC_50_ value of 65–80 μM, ∼10,000-fold less potent than nicotine at 7.53 nM. Complete inhibition of ^125^I-a-conotoxinMII or [^125^I]A-85380 binding by cotinine occurred at ∼ 1 mM. This study suggested that cotinine might be more potent at α3/α6β2^∗^ than α4β2^∗^ receptors (O’Leary et al., 2008).

In cultured bovine chromaffin cells, EC_50_ values were 130 μM for cotinine and 0.3 μM for nicotine for displacing high-affinity [^3^H]epibatadine binding, and were 310 μM for cotinine and 1.6 μM for nicotine for displacing low-affinity [^3^H]epibatidine binding (Vainio and Tuominen, 2001). In Torpedo membrane, Ki values for displacing [^3^H]nicotine were 520 μM for cotinine and 310 nM for nicotine (Abood et al., 1981). Another study reported that IC_50_ values for inhibiting [^3^H]nicotine binding were 200 mM for cotinine and 0.5 mM for nicotine. IC_50_ values for displacing [^125^I]α-bungarotoxin binding were 50 μM for cotinine and 25 μM for nicotine. In these assays, cotinine appeared to be only 50% efficacious compared to nicotine (Riah et al., 1999). These studies suggested that cotinine might have greater potency at low-affinity α7 nAChRs in Torpedo membrane.

In cultured *Xenopus* oocytes or Chinese hamster ovary (CHO) cells expressing human α7 nAChRs, cotinine at concentrations up to 1 mM did not elicit appreciable activation of these receptors (Briggs and McKenna, 1998; Terry et al., 2015a; Alijevic et al., 2020). However, cotinine functioned as a full agonist of a mutant human α7 nAChR (α7V274T) with its EC_50_ value at 70 μM (Briggs et al., 1999). In cultured *Xenopus* oocytes, cotinine up to 100 μM didn’t activate human α4β2 nAChRs (Terry et al., 2015a). On the other hand, cotinine activated human α4β2 nAChRs with EC_50_ value at ∼90 μM in CHO cells, and cotinine was ∼115 fold less potent and 40% less efficacious than nicotine, suggesting a weak partial agonist activity of cotinine on α4β2 nAChRs (Alijevic et al., 2020). Pretreatment with cotinine up to 100 μM did not alter acetylcholine-induced currents in either α7 or α4β2 nAChRs, whereas short term cotinine incubation increased acetylcholine-induced currents in α7, but not α4β2 receptors, suggesting that short-term exposure to cotinine upregulated acetylcholine activation of α7 receptors (Terry et al., 2015a). On the other hand, cotinine was shown to inhibit acetylcholine-elicited response in human α7 nAChRs with IC_50_ values at 175 μM; cotinine was ∼250 fold less potent than nicotine, but similarly efficacious to nicotine (Briggs and McKenna, 1998).

These studies revealed a complex landscape of the interaction between cotinine and nAChRs, with cotinine functioning mainly as a weak agonist of α3/α6β2^∗^, α4β2^∗^, and α7 nAChRs. In addition, the potency and efficacy of cotinine appear to be influenced by subunit compositions of nAChRs. Interestingly, α7 and β2 subunits can form functional α7β2 heteromeric nAChRs in the brain (Wu et al., 2016). Whether the α7β2 nAChRs would interact with cotinine remains to be determined. More importantly, most of these studies reveal that the potency values of cotinine greatly exceed the physiological levels of cotinine obtained in smokers, suggesting that nAChRs may not be the main target of cotinine in smokers. However, more research will be needed to identify receptors that cotinine can interact with at physiological levels.

Cotinine did not show significant binding to serotonin receptors (Fuxe et al., 1979), muscarinic receptors (Anderson and Arneric, 1994), or NMDA receptors (Aizenman et al., 1991). A recent study was in line with these findings (Terry et al., 2015a). In addition, cotinine at 10 μM was found to lack significant binding to or action on more than 70 molecular targets, including major neurotransmitter receptors and transporters (adenosine, adrenergic, dopamine, GABA, glutamate, glycine, histamine, muscarinic, opioid, serotonin, sigma 1 and 2), ion channels (Ca^2+^, K^+^, Na^+^), second messengers (e.g., nitric oxide), prostaglandins (e.g., leukotriene and thromboxane), brain/gut peptides (e.g., angiotensin II, bradykinin, endothelin, neurokinin, neuropeptide), and enzymes (acetylcholine esterase, phosphodiesterase, protein kinase A and C) (Terry et al., 2015a).

Interestingly, one study reported the isolation of a putative cotinine receptor from rat brain. This 40-kDa protein had greater affinity for cotinine than for α-bungarotoxin, nicotine and acetylcholine, with IC_50_ values at 0.19 μM, 1.7 μM, 110 μM, and 160 mM, respectively. Amino acid sequence analysis of this protein showed no identity to then known proteins except for the homology to the human p205 synovial fluid protein (Riah et al., 2000). A recent study demonstrated that both cotinine and nicotine bound with similar affinity (∼10–20 μM) to the myeloid differentiation protein 2, an accessory protein of Toll-like receptor 4, to regulate glia-mediated neuroinflammation in a nAChRs-independent manner (Li et al., 2021). These studies suggest that cotinine may function through non-nAChRs-mediated mechanisms. However, whether these mechanisms may underlie cotinine’s physiological effects remains to be determined.

## Neuropharmacological Effects of Cotinine

Several studies indicated that cotinine altered serotonin turnover in the brain. Chronic exposure of rats to cotinine in drinking water increased daily urinary excretion of 5-hydroxyindoleacetic acid, the major metabolite of serotonin, suggesting that cotinine might alter serotonin turnover (De Clercqm and Truhaut, 1963). Systemic administration of cotinine in mice significantly increased tissue content of serotonin and 5-hydroxyindoleacetic acid in mesencephalon and diencephalon, but not in the cerebral cortex. The effect of cotinine on serotonin levels was similarly robust to nicotine in mesencephalon, but less robust in diencephalon. In contrast, elevation of 5-hydroxyindoleacetic acid was more pronounced following cotinine treatment in both regions (Essman, 1973). Repeated intraperitoneal injections of cotinine attenuated α-propyldopacetamide-induced cortical serotonin depletion to a similar degree as nicotine treatment, which was not altered by mecamylamine pretreatment (Fuxe et al., 1979). In addition, low concentrations of cotinine, but not nicotine, reduced serotonin uptake, and increased spontaneous serotonin release *in vitro* in neocortical slices (Fuxe et al., 1979).

Cotinine can increase brain dopamine transmission. In rat striatal slices or minces, cotinine increased [^3^H]dopamine overflow in concentration-, Ca^2+^-, and nAChRs-dependent manners, with EC_50_ values ranging from 30 to 350 μM (Dwoskin et al., 1999; Oliver et al., 2007). This increase appears to be due mainly to facilitated synaptic dopamine release, but not dopamine uptake (Dwoskin et al., 1999). Cotinine was ∼1000 fold less potent than nicotine, but was as fully efficacious as nicotine (Oliver et al., 2007). In squirrel monkeys, cotinine stimulated [^3^H]dopamine release from striatal synaptosomes through both α3/α6β2^∗^ and α4β2^∗^ nAChRs-dependent mechanisms. EC_50_ values were 270 and 500–750 μM for α3/α6β2^∗^- and α4β2^∗^-mediated release, respectively, which were 200–750 fold less potent than nicotine. Cotinine was equally efficacious to nicotine in producing α4β2^∗^-mediated release, but was only 50% efficacious in inducing α3/α6β2^∗^-mediated release in the medial caudate (O’Leary et al., 2008).

Cotinine has also been shown to alter peripheral catecholamine activity. Cotinine induced concentration-dependent depolarization of mouse sympathetic superior cervical ganglion, which may lead to altered catecholamine release. Cotinine was ∼80 fold less potent than nicotine (Schroff et al., 2000). In isolated rabbit heart, cotinine inhibited [^3^H]norepinephrine release evoked by sympathetic nerve stimulation, whereas nicotine increased stimulated release of [^3^H]norepinephrine (Chahine et al., 1993). In cultured bovine adrenal chromaffin cells, cotinine increased the release of [^3^H]noradrenaline, which was accompanied by increased protein kinase C expression and phorbol dibutyrate binding. Cotinine was less potent than nicotine in inducing these effects (Vainio et al., 1998b). In isolated rat adrenal gland, cotinine inhibited catecholamine release evoked by high calcium and acetylcholine, but not by high K^+^, whereas nicotine produced biphasic effect on catecholamine release induced by acetylcholine and high K^+^. Both cotinine and nicotine depressed catecholamine release evoked by activation of nicotinic or M1 muscarinic receptors (Koh et al., 2003).

Cotinine affected extracellular amino acid levels in the brain. Perfusion of striatum with cotinine via reversed microdialysis in rats decreased the levels of aspartic acid, serine, and glutamine, but did not change the levels of glutamic acid, glycine, taurine, or threonine (Toth et al., 1993).

These studies suggest that cotinine can alter neurochemistry in the nervous system, especially monoamine neurotransmission. However, it remains unknown how these cotinine-induced neurochemical changes may contribute to the effects of cotinine on behavior. Given the important role of monoamine neurotransmitters, especially dopamine, in nicotine reinforcement and the development of nicotine addiction (De Biasi and Dani, 2011), it will be interesting to determine potential roles of cotinine in nicotine reinforcement and use.

## Neuroprotective Effects of Cotinine

In cultured PC12 cells or rat primary cortical neurons, cotinine attenuated loss in cell viability induced by growth factor withdrawal, Aβ_1__–__42_ incubation, and excessive glutamate, with the (−) isomers more effective than the (+) isomers. In these effects, cotinine showed similar potency and efficacy to nicotine (Buccafusco and Terry, 2003; Terry et al., 2005; Burgess et al., 2012; Gao et al., 2014). Cotinine was shown in *in vitro* studies to bind to Aβ_1__–__40_ peptides, and to inhibit Aβ_1__–__42_ peptide precipitation and aggregation with similar affinity (Ka ∼10 nM) and efficacy to nicotine (Salomon et al., 1996; Szymańska et al., 2007; Echeverria et al., 2011). Cotinine increased neurotrophic factors level, and activated pro-survival signaling markers (Sadigh-Eteghad et al., 2020). In addition, cotinine attenuated 6-hydroxydopamine-induced cytotoxicity (a Parkinson’s disease model) in cultured human neuroblastoma cells. Cotinine was equally effective to nicotine at a lower concentration of 6-hydroxydopamine, but less effective than nicotine at a higher concentration of 6-hydroxydopamine (Riveles et al., 2008). Cotinine was shown to increase total antioxidant capacity and reduce oxidative stress. Cotinine reduced O_2_ consumption, H_2_O_2_ accumulation, and the production of oxygen free radicals to the similar degree as nicotine (Srivastava et al., 1989; Soto-Otero et al., 2002; Sadigh-Eteghad et al., 2020). Cotinine also attenuated production of pro-inflammatory cytokines and increased levels of anti-inflammatory cytokines (Rehani et al., 2008; Bagaitkar et al., 2012; Sadigh-Eteghad et al., 2020). These anti-oxidative stress, anti-inflammatory, and pro-survival effects of cotinine may contribute to the neuroprotective effects of cotinine, suggesting potential beneficial effects of cotinine in neurodegenerative conditions such as Alzheimer’s disease and Parkinson’s disease.

## Behavioral Effects of Cotinine

### Effects of Cotinine on Locomotor Activity

Low doses of cotinine were shown to alter locomotor activity, with one study reporting reduced (Wiley et al., 2015), and another demonstrating increased locomotor activity (Wang et al., 2020b). Interestingly, low doses of nicotine produced biphasic effects with initial decrease followed by subsequent increase of locomotor activity (Wiley et al., 2015). These findings suggest differential effects of low doses of cotinine and nicotine on locomotor activity. Repeated daily treatment with cotinine decreased locomotor activity overtime in adult, but not adolescent rats, suggesting age-dependent effects (Marusich et al., 2017). Chronic cotinine treatment decreased locomotor activity in mice receiving chronic restraint stress, but not in non-stressed mice, suggesting an interaction between cotinine and stress on locomotor activity (Grizzell et al., 2014a).

Cotinine was shown to alter motor function induced by either nicotine or ethanol. Adding a low dose of cotinine to nicotine solution enhanced the locomotor-stimulating effect of nicotine (Clemens et al., 2009). Intra-ventricular or intra-cerebellar administration of cotinine or nicotine attenuated ethanol-induced motor incoordination in mice, with cotinine producing less robust effects than nicotine. The nAChR antagonists, hexamethonium and trimethaphan, blocked these effects of cotinine. Cotinine and nicotine also antagonized effects of adenosine agonists on ethanol-induced motor incoordination. These data suggest an interaction between nicotinic cholinergic and adenosinergic systems within the cerebellum, and its involvement in modulating ethanol-induced motor incoordination (Dar et al., 1993, 1994).

### Effects of Cotinine on Conditioning-Related Behaviors

A series of studies by Goldberg and colleagues indicated that cotinine altered schedule-controlled, food-conditioned responding in a species-, schedule-, and dose-dependent manner. In dogs, cotinine decreased response rates during a fixed-ratio (FR) and a fixed-interval (FI) schedule, whereas nicotine decreased response rates during the FR schedule, but produced biphasic effects during the FI schedule (Risner et al., 1985). In squirrel monkeys, cotinine reduced overall responses during the FR schedule, and produced biphasic effects during the FI schedule. Nicotine produced biphasic effects during both schedules (Risner et al., 1985). In rats, cotinine dose-dependently increased response rate during a FI, but not a FR schedule. Nicotine produced biphasic effects during the FI schedule, but only decreased response rates during the FR schedule (Goldberg et al., 1989). Interestingly, the effects of nicotine, but not cotinine, were antagonized by the pretreatment with the non-selective nAChRs antagonist mecamylamine in rats (Goldberg et al., 1989). These studies suggest that cotinine may alter reinforcement-related behavior.

Several studies indicated that cotinine could be substituted for nicotine in producing nicotine-like discriminative stimulus effects. Intra-ventricular administration of cotinine fully generalized to nicotine in inducing discriminative stimulus effects in rats trained on nicotine under a variable-interval schedule in a training dose-dependent manner (Rosecrans and Chance, 1977; Rosecrans et al., 1978). Later studies by Goldberg and colleagues demonstrated that systemic cotinine was nearly completely substituted for nicotine in both rats and squirrel monkeys; EC_50_ value of cotinine was approximately 30 mg/kg and cotinine was 1000-2000 fold less potent than nicotine (Goldberg et al., 1989; Takada et al., 1989). These authors noted that there was up to 0.1% of nicotine as impurity in continine, which was speculated to contribute to effects of cotinine. However, no pharmacological or blood nicotine data were provided to support this speculation. These authors also found that cotinine’s effects on food-reinforced behaviors were insensitive to nAChR blockade with mecamylamine (Goldberg et al., 1989). Therefore, the potential confound with nicotine impurity remains unproven.

In a recent study, we demonstrated that cotinine was self-administered intravenously in rats in a dose-dependent manner (Ding et al., 2021). Rats acquired self-administration of cotinine over time and responded more on an active lever than an inactive lever. Cotinine induced more infusions and greater breakpoints than vehicle under both fixed-ratio and progressive-ratio schedules. The comparison between cotinine and nicotine revealed similarities and differences in self-administration which were dependent on reinforcement schedule and dose. In general, cotinine self-administration was less robust than that of nicotine. In addition, this study found that pharmacological manipulation of nAChRs with mecamylamine and varenicline only reduced nicotine, but not cotinine, self-administration, suggesting differential involvement of nAChRs in cotinine and nicotine self-administration. It remains unknown how cotinine may contribute to nicotine self-administration.

## Effects of Cotinine on Neuropsychiatric Symptoms

The neuropsychiatric disease schizophrenia is characterized by impairment in sensorimotor gating. Schizophrenia is comorbid with high rates of tobacco smoking and it has been proposed that nicotine can provide protective effects against neuropsychiatric symptoms in schizophrenia (Lucatch et al., 2018). Pre-pulse inhibition (PPI) of the acoustic startle reflex is a widely used experimental model for schizophrenia. The PPI paradigm for measuring sensorimotor gating measures suppression of the amplitude of a startle reflex to a startling stimulus when it is preceded by a weaker pre-pulse stimulus (Geyer et al., 2001). In a series of studies, deficits in PPI was induced in rats by the non-selective dopamine receptor agonist apomorphine, the non-competitive NMDA receptor antagonist MK-801, and non-specific muscarinic receptor antagonist scopolamine. Pretreatment with cotinine reversed deficits in PPI induced by these compounds (Buccafusco and Terry, 2003; Terry et al., 2005). In addition, in DBA/2 mice exhibiting spontaneous deficits in hippocampal sensory inhibition, both acute and chronic cotinine increased conditioning amplitude in a hippocampal sensory inhibition test, suggesting that cotinine may attenuate deficits in sensory inhibition (Wildeboer-Andrud et al., 2014). These results suggest that cotinine may be beneficial for schizophrenia and other sensory gating disorders.

Post-traumatic stress disorder (PTSD) is an anxiety disorder triggered by exposure to life-threatening traumatic events. Epidemiological studies indicate a high prevalence of tobacco use in PTSD patients, and tobacco use has been proposed as a form of self-medication to improve neuropsychiatric symptoms in PTSD (Leonard et al., 2001). In rodent fear conditioning models of PTSD, cotinine administration, either systemically or locally into the hippocampus and medial prefrontal cortex, reduced the retention of fear memory and facilitated extinction of fear memory (Zeitlin et al., 2012; Aguiar et al., 2013; Alvarez-Ricartes et al., 2018; Oliveros-Matus et al., 2020). Interestingly, the effects of cotinine in the hippocampus were shared by nicotinic antagonists, such as mecamylamine, dihydro-β-erythroidine, and methyllycaconitine, suggesting that inhibition of nAChRs may underlie cotinine’s effects in hippocampus (Aguiar et al., 2013). Co-administration of methyllycaconitine but not dihydro-β-erythroidine, with cotinine into the medial prefrontal cortex abolished the effect of cotinine on extinction of fear conditioning, suggesting an involvement of α7 nAChRs-, but not α4β2^∗^ nAChRs-mediated mechanisms within the medial prefrontal cortex (Oliveros-Matus et al., 2020). In addition, systemic cotinine reduced anxiety-like behaviors in the elevated plus maze test and the open field test following fear conditioning. These effects of cotinine were accompanied by an increase of phospho-ERK1/2 (Zeitlin et al., 2012; Aguiar et al., 2013), calcineurin (Alvarez-Ricartes et al., 2018), and GFAP + immunoreactivity (Oliveros-Matus et al., 2020) in hippocampus and PFC. These findings suggest that cotinine may have therapeutic potential for PTSD-like symptoms.

Cotinine reduced depressive-like behaviors induced by chronic stress (Grizzell et al., 2014a, b; Perez-Urrutia et al., 2017), fear conditioning (Alvarez-Ricartes et al., 2018), chemotherapy (Iarkov et al., 2016), and the development of Alzheimer’s disease (Patel et al., 2014). These effects of cotinine were associated with increase of vascular endothelial growth factor, pAKT-GSK3β phosphorylation, synaptic density and PSD95 expression, and calcineurin in the hippocampus and/or prefrontal cortex (Grizzell et al., 2014a, b; Patel et al., 2014; Alvarez-Ricartes et al., 2018). These results suggest that cotinine may provide beneficial effects for depression.

## Effects of Cotinine on Cognitive Functions

It is well known that nicotine can enhance cognitive functions (Valentine and Sofuoglu, 2018), and recent preclinical findings indicate that cotinine can also provide cognitive benefits. In a series of studies using the delayed matching-to-sample task to measure working memory and attention in Macaques, cotinine was shown to increase the overall task accuracy by itself, and produced persistent attenuation of ketamine- and distractor-induced impairment in task accuracy (Buccafusco and Terry, 2003, 2009; Terry et al., 2005). Cotinine improved sustained attention in rats tested in the five choice serial reaction time task. The non-competitive NMDA receptor antagonist MK-801 reduced overall accuracy rate, increased impulsive- and compulsive-like behaviors, and caused cognitive inflexibility. Both acute and chronic cotinine significantly attenuated MK-801-induced impairments in task accuracy, and reduced impulsive- and compulsive-like behaviors (Terry et al., 2012). In Swiss mice, cotinine suppressed the scopolamine-induced deficit in short-term spatial memory in Y-maze test, and its effects were less efficacious than those of nicotine (Callahan et al., 2021). These studies suggest that cotinine may have therapeutic potential for neuropsychiatric disorders by improving attention and memory, especially those characterized by alterations in glutamate and cholinergic neurotransmission.

Repeated cotinine improved spatial recognition memory in a novel location recognition test in rats receiving chemotherapy (Iarkov et al., 2016). Chronic cotinine treatment improved working memory performance in the radial arm water maze test (Grizzell et al., 2014a), and reversed the deficit in visual recognition memory in the novel object recognition test after prolonged restraint stress in mice (Grizzell et al., 2014a; Perez-Urrutia et al., 2017; Mendoza et al., 2018b). These behavioral changes were accompanied by normalization of the number and arborization of GFAP + cells (Perez-Urrutia et al., 2017; Mendoza et al., 2018b), increases in GSK3β phosphorylation, and enhancement of synaptic density in prefrontal cortex and hippocampus (Grizzell et al., 2014a).

Cotinine attenuated age- and neurodegeneration-related cognitive impairments. In senescent mice, chronic cotinine treatment reversed impairments in spatial and recognition learning and memories in the Morris water maze and novel object recognition tasks in a α7 nAChR-dependent manner (Sadigh-Eteghad et al., 2020). In both transgenic and Aβ-induced models of Alzheimer’s disease, chronic cotinine administration prevented working and reference memory impairments, and improved cognitive performance in several learning and memory tasks, including circular platform, radial arm water maze, Y-maze, and cognitive interference task. Cotinine also restored short-term visual recognition memory performance in a novel object recognition test. Such a protective effect was not observed in the Morris water maze or platform recognition task, suggesting a task-dependent effect (Echeverria et al., 2011; Grizzell et al., 2017; Boiangiu et al., 2020). These beneficial effects were associated with reduction in Aβ, p-Tau, neuroinflammation, and acetylcholinesterase activity, as well as increase in neurotrophic factors, total antioxidant capacity, pro-survival signaling, and synaptic plasticity in hippocampus and/or prefrontal cortex (Echeverria et al., 2011; Patel et al., 2014; Grizzell et al., 2017; Boiangiu et al., 2020, 2021). All these factors may converge to promote neuronal synaptic plasticity and long-term potentiation, inhibit neuronal cell death, and improve memory and attention.

Cotinine also improved cognitive performance in Fmr1^–/–^ mice, a murine model of Fragile X syndrome. Cotinine rescued deficits in spatial memory in the coordinate and categorical spatial processing tests, increased the performance toward a novel object in the novel object recognition test, and reversed memory impairment in the temporal order memory test. This study also established the causal role of the AKT-GSK3β signaling pathway in mediating cotinine’s effects by demonstrating that cotinine failed to enhance cognition in GSK3β knockin mice that exhibited impaired phosphorylation of GSK3β (Pardo et al., 2017).

## Effects of Cotinine in Humans

Cotinine at doses producing blood levels up to 3000 ng/ml (∼17 μM) appeared to be safe and well-tolerated in humans, with no major side effects other than dizziness and headache (Bowman and McKennis, 1962; Hatsukami et al., 1997, 1998a). Cotinine had no appreciable cardiovascular effects, did not alter heart rate, systolic or diastolic blood pressure, or electrocardiogram in healthy non-smokers (Zevin et al., 1997, 2000; Herzig et al., 1998) or abstinent smokers (Benowitz et al., 1983b; Keenan et al., 1994). Cotinine didn’t change skin temperature, weight, or caloric intake (Benowitz et al., 1983b; Hatsukami et al., 1997, 1998b). Cotinine didn’t appear to alter mood state in healthy non-smokers (Herzig et al., 1998; Zevin et al., 2000). Discontinuation from repeated cotinine administration did not induce drug-like effects or withdrawal-like symptoms (Hatsukami et al., 1997). Therefore, cotinine appears to have a more favorable toxicology profile compared to nicotine.

There is evidence that cotinine alters withdrawal-related psychological and physiological signs and symptoms. Acute cotinine infusion in abstinent smokers reduced self-reported desire to smoke, irritability, low energy, anxiety and tension. A tendency to think less about smoking was also observed. These effects were small and subtle, and there was no placebo control group included for a comparison (Benowitz et al., 1983b). One randomized, double-blind, placebo-controlled, counterbalanced study examined the effects of cotinine on symptoms related to acute smoking cessation. Compared to placebo, cotinine slightly increased the self-ratings of “pleasant” and “sedated,” but reduced the self-ratings of “restless,” “anxious/tense,” “insomnia,” suggesting cotinine alterations of subjective ratings during acute withdrawal (Keenan et al., 1994). On the other hand, a sebsequent study reported that cotinine increased “restless” and “impatience”, and as dose increased, tended to increase then decrease “depressed mood” and “difficulty concentrating” during acute withdrawal (Schuh et al., 1996). Another study demonstrated that cotinine caused a greater severity of “difficulty concentrating,” an increase in fatigue, and potentially less abstinence in abstinent smokers compared to nicotine patch and nicotine plus cotinine treatments. In addition, cotinine completely eliminated nicotine patch’s effects on reducing withdrawal symptoms (Hatsukami et al., 1998b). Cotinine did not alter the self-reported number of cigarettes smoked, the average weights of the collected cigarette butts, or alveolar carbon monoxide levels in current smokers (Hatsukami et al., 1998a).

Cotinine was shown to cause cognitive deficit in healthy non-smokers. Cotinine significantly impaired memory on the long list of a verbal recall task, and slowed serial information processing in a visual choice reaction time task (Herzig et al., 1998). On the other hand, cotinine did not alter cognitive function in several attention-related tasks, including the Symbol Digit Modalities test, the Stroop test, and Letter Cancellation test (Hatsukami et al., 1998b). These different results suggest that effects of cotinine on cognitive function may be task-dependent. Given the cognition-enhancing effects of cotinine in animal models of various neuropsychiatric and neurodegenerative diseases (Terry et al., 2005; Echeverria and Zeitlin, 2012; Mendoza et al., 2018a), it will be interesting to examine the potential effects of cotinine on cognitive function in these disease states.

In these studies, cotinine was administered either acutely or repeatedly over a short period of time, usually fewer than 14 days. Given the chronic relapsing nature of habitual smoking, it would be worth examining effects of cotinine over a longer-term administration period to better understand the chronic effects of cotinine in humans.

## Potential Mechanisms Underlying Cotinine’s Effects

### Potential Involvement of Nicotinic Acetylcholine Receptors?

Since cotinine has been shown to be a weak agonist of nAChRs, most studies have focused on determining whether nAChRs could mediate the effects of cotinine. There are a number of studies indicating that certain effects of cotinine are dependent on activation of nAChRs. Cotinine blunted pain perception and this was blocked by mecamylamine (Erenmemisoglu and Tekol, 1994). Cotinine increased phorbol binding and intracellular Ca^2+^ concentrations in cultured bovine adrenal chromaffin cells, and these effects were antagonized by nAChR antagonists, hexamethonium, chlorisondamine, and dihydro-β-erythroidine (Vainio et al., 1998b, 2000). Cotinine-increased striatal dopamine overflow was attenuated by mecamylamine and dihydro-β-erythroidine (Dwoskin et al., 1999; Oliver et al., 2007). Cotinine-attenuated production of pro-inflammatory cytokines was reversed by α-bungarotoxin (Rehani et al., 2008; Bagaitkar et al., 2012). Dihydro-β-erythroidine and α-bungarotoxin prevented cotinine-induced reversal of sensory inhibition deficits in mice (Wildeboer-Andrud et al., 2014). Methyllycaconitine abolished the effect of cotinine on extinction of fear conditioning (Oliveros-Matus et al., 2020) and age-related cognitive impairments (Sadigh-Eteghad et al., 2020). Dihydro-β-erythroidine, but not methyllycaconitine, co-infusion abolished cotinine’s effect on GFAP (Oliveros-Matus et al., 2020). The preponderance of evidence, therefore, suggests that the effects of cotinine in these physiological domains may be mediated through nAChRs.

There is also evidence, however, suggesting that certain effects of cotinine are not mediated by nAChRs. Effects of cotinine on cortical serotonin depletion and on food-reinforced operant responding were not antagonized by mecamylamine (Fuxe et al., 1979; Goldberg et al., 1989). The nAChR antagonist hexamethonium reduced nicotine toxicity, but enhanced cotinine toxicity (Riah et al., 1999). Cotinine alters BBB permeability of saquinavir and sucrose, and these effects are not altered by α-bungarotoxin, methyllycaconitine, or mecamylamine (Abbruscato et al., 2002; Manda et al., 2010). The effects of cotinine on Aβ-induced cell death was not affected by mecamylamine (Burgess et al., 2012). Cotinine self-administration in rats were not altered by mecamylamine or varenicline (Ding et al., 2021). Furthermore, the inhibitory effects of cotinine on lipopolysaccharide-induced pro-inflammatory factors were not affected by either mecamylamine or RNAi-mediated down-regulation of α7 nAChRs (Li et al., 2021).

These differences may be driven by the varying experimental systems implemented in these studies. In addition, they suggest that cotinine may act through both nAChRs- and non-nAChR-mediated mechanisms, which echoes findings implicating cotinine’s binding and interactions with both nAChRs and other protein targets reviewed above. However, these non-nAChRs mechanisms remain to be further characterized.

### Cotinine as a Nicotinic Acetylcholine Receptors Desensitizing Agent?

Cotinine has been proposed as a desensitization agent for nAChRs (Buccafusco et al., 2009). There are several lines of evidence supporting this hypothesis. First, cotinine increased dopamine overflow from rat striatal slices; the increase peaked shortly after cotinine superfusion, but gradually diminished during continued cotinine incubation, suggesting the development of receptor desensitization overtime (Dwoskin et al., 1999). Second, cotinine pretreatment diminished several effects mediated by the activation of nAChRs, including nicotine-induced increase of intracellular Ca^2+^ concentrations and norepinephrine release in cultured bovine chromaffin cells (Vainio et al., 1998a, 2000), nicotine-induced dopamine release from striatal minces (Oliver et al., 2007) and nucleus accumbens (Sziraki et al., 1999), nicotine-stimulation of mouse sympathetic superior cervical ganglion neurons (Schroff et al., 2000), acetylcholine-stimulated catecholamine release from adrenal gland (Koh et al., 2003), and ganglionic stimulant-mediated increase of arterial blood (Buccafusco et al., 2007). Third, cotinine treatment upregulated protein expression of α4β2 nAChRs, and favored the assembly of high sensitivity (α4)_2_(β2)_3_ stoichiometry on plasma membrane of cultured undifferentiated mouse neuroblastoma 2a cells (Fox et al., 2015), consistent with desensitization-induced upregulation of nAChRs (Henderson and Lester, 2015).

On the other hand, there is also evidence which doesn’t support cotinine as a nAChR desensitizer. First, several studies indicate that cotinine pretreatment does not alter effects mediated by activation of nAChRs; these effects include the inhibitory effects of nicotine on high voltage spindles in electroencephalographic recording in rats (Radek, 1993), acetylcholine-induced currents in human α7 or α4β2 nAChRs expressed in oocytes (Terry et al., 2015a), or nicotine’s effects on locomotor activity or ultrasonic vocalization (Wang et al., 2020b). Second, cotinine pretreatment enhanced acetylcholine-induced currents in human α7 nAChRs expressed in oocytes, implicating cotinine as a α7 nAChR sensitizer (Terry et al., 2015a, b). Third, chronic cotinine treatment reduced both high-affinity [^3^H]epibatidine and low-affinity [^125^I]α-bungarotoxin binding in various rat brain regions (Buccafusco and Terry, 2003; Terry et al., 2005), and α6β2β3 receptor density in mouse neuroblastoma 2a cells (Fox et al., 2015), which is inconsistent with the up-regulation of nAChRs induced by desensitization (Henderson and Lester, 2015). Interestingly, chronic cotinine treatment also reduced M_2_ muscarinic acetylcholine receptor binding in several rat brain regions, although the importance of these changes remains unknown (Terry et al., 2005).

### Cotinine as a Potential Positive Allosteric Modulator of α7 Nicotinic Acetylcholine Receptors?

Cotinine has also been hypothesized to be a potential Positive Allosteric Modulator (PAM) of α7 nAChRs to explain its behavioral effects in various animal models involving cognitive impairments (Grizzell and Echeverria, 2015; Echeverria et al., 2016; Oliveros-Matus et al., 2020). Currently, there is no direct evidence supporting this hypothesis. One study reported that sustained exposure to cotinine at 1 μM, but not 0.1 or 10 μM potentiated acetylcholine-induced currents in human α7 nAChRs expressed in *Xenopus* oocytes, which may partially support this proposal (Terry et al., 2015a). It is noted that the potentiation effects of cotinine occurred only after 8 min of exposure, but not after shorter exposure, raising the possibility of cotinine as a sensitizer of α7 nAChRs (Terry et al., 2015b). An earlier study found that cotinine inhibited acetylcholine-mediated currents in α7 nAChRs expressed in *Xenopus* oocytes with the IC_50_ value at ∼175 μM (Briggs and McKenna, 1998). In addition, cotinine at 3.7-33.3 μM inhibited acetylcholine-mediated currents in α7 nAChRs expressed in Chinese hamster ovary cells, but not in *Xenopus* oocytes (Alijevic et al., 2020). Therefore, more evidence will be needed for this hypothesis.

## Future Directions

Most studies have focused on the interactions between cotinine and α4β2^∗^ and α7 nAChRs, and few studies have examined potential involvement of other nAChR subtypes. For example, cotinine was shown to be more potent toward α3/α6β2^∗^ than α4β2^∗^ nAChRs in monkey striatum (O’Leary et al., 2008). Moreover, cotinine treatment down-regulated α6β2β3^∗^ receptor density in mouse neuroblastoma 2a cells (Fox et al., 2015). Therefore, it will be important to study binding and interaction profiles of cotinine for other nAChR subtypes to provide more insights into the mechanisms involved in cotinine’s effects.

Since cotinine is a weak agonist at nAChRs, many studies employed relatively high concentrations of cotinine to study its effects (Goldberg et al., 1989; Takada et al., 1989; Vainio et al., 1998a, 2000; Oliver et al., 2007). Although these results provided valuable information, cotinine concentrations used in these studies were greatly higher than blood cotinine levels attained in habitual smokers. In addition, it was noted that potential contamination of cotinine samples with small amount of nicotine as an impurity confounded the interpretation of some studies (Goldberg et al., 1989; Schroff et al., 2000). Some recent studies applied cotinine at doses yielding blood cotinine levels close to physiological levels, and reported various effects of cotinine (Terry et al., 2005, 2012; Echeverria et al., 2011; Grizzell et al., 2014a; Ding et al., 2021). Therefore, it will be imperative to study cotinine’s effects with cotinine concentrations at or close to the physiological levels in smokers to increase translational value of the findings. In addition, given the chronic nature of smoking, it is highly valuable to investigate long term adaptive changes within the brain at molecular, cellular, and circuit levels following chronic cotinine exposure at these physiological levels. Such information has potential translational significance and may shed light on the development of therapeutic strategy targeting cotinine and its effects.

Another remaining question is whether cotinine can contribute to the development of nicotine use, abuse and addiction. Our recent study (Ding et al., 2021) indicates that cotinine supported intravenous self-administration in rats, suggesting that cotinine may be reinforcing by itself. These reinforcing effects of cotinine may play a role in nicotine reinforcement. Therefore, it will be interesting to determine how cotinine may alter nicotine reinforcement. Such studies will provide valuable evidence supporting potential therapeutic value of targeting cotinine and its effects for treating nicotine addiction.

## Author Contributions

Z-MD conceptualized and drafted the manuscript. KV and XT contributed to the draft and review of the manuscript. All authors approved the final manuscript.

## Conflict of Interest

The authors declare that the research was conducted in the absence of any commercial or financial relationships that could be construed as a potential conflict of interest.

## Publisher’s Note

All claims expressed in this article are solely those of the authors and do not necessarily represent those of their affiliated organizations, or those of the publisher, the editors and the reviewers. Any product that may be evaluated in this article, or claim that may be made by its manufacturer, is not guaranteed or endorsed by the publisher.
